# Protracted Effects of Juvenile Stressor Exposure Are Mitigated by Access to Palatable Food

**DOI:** 10.1371/journal.pone.0096573

**Published:** 2014-05-06

**Authors:** Jennifer Christine MacKay, Jonathan Stewart James, Christian Cayer, Pamela Kent, Hymie Anisman, Zul Merali

**Affiliations:** 1 School of Psychology, University of Ottawa, Ottawa, Ontario, Canada; 2 Department of Psychiatry, University of Ottawa, Ottawa, Ontario, Canada; 3 Department of Cellular and Molecular Medicine, University of Ottawa, Ottawa, Ontario, Canada; 4 Institute of Neuroscience, Carleton University, Ottawa, Ontario, Canada; 5 University of Ottawa Institute of Mental Health Research, Ottawa, Ontario, Canada; University of Minnesota, United States of America

## Abstract

Stressor experiences during the juvenile period may increase vulnerability to anxiety and depressive-like symptoms in adulthood. Stressors may also promote palatable feeding, possibly reflecting a form of self-medication. The current study investigated the short- and long-term consequences of a stressor applied during the juvenile period on anxiety- and depressive-like behavior measured by the elevated plus maze (EPM), social interaction and forced swim test (FST). Furthermore, the effects of stress on caloric intake, preference for a palatable food and indices of metabolic syndrome and obesity were assessed. Male Wistar rats exposed to 3 consecutive days of variable stressors on postnatal days (PD) 27–29, displayed elevated anxiety-like behaviors as adults, which could be attenuated by consumption of a palatable high-fat diet. However, consumption of a palatable food in response to a stressor appeared to contribute to increased adiposity.

## Introduction

Adolescent and childhood obesity has become a worldwide epidemic to the extent that globally, approximately 200 million school aged children can be classified as either overweight or obese [1]. Further, childhood and adolescent obesity is a strong predictor of adult obesity [2]–[4], and have been associated with adverse long-term health outcomes such as, hypercholesterolemia, insulin resistance [5], hypertension [6], type-2 diabetes [7], nonalcoholic fatty liver disease [8] and various cancers [9], [10].

Recent increases in the prevalence and incidence of obesity have been attributed to the interplay of a variety of different factors, such as genetics, the family environment, levels of physical activity, advertising, and sedentary behaviors [11]. Of particular interest is the role of stress in the development and maintenance of obesity, as the level of daily stressors individuals have been experiencing continues to increase [12]–[14]. In this regard, a stressor-induced preference for palatable foods (especially those with a high fat and/or sugar content) has been documented in both animals and humans [15]–[18]. This preference was proposed to serve as a form of self-medication to protect against the adverse effects of stress [19]. Access to a palatable food has been shown to protect against behavioral disturbances elicited by inescapable foot shock [20], reduce sympathetic responses to a stressor [21]–[30], and diminish hypothalamic-pituitary adrenal (HPA) axis activity [31]–[34]. Consumption of palatable foods may also limit some of the long-term negative effects of an early life stressor (maternal separation) [35].

Although self-medication with a palatable food can have some beneficial effects on psychological functioning, it may be a counterproductive long-term stress coping strategy. The excess calories gained from consumption of a high fat and/or sugar diet leads to an increase in adipose tissue [36], which secretes several hormones and signaling factors [37]–[39] involved in the regulation of food intake [37], [40]. Increases of adipose tissue and the resulting endocrinological consequences have been implicated in atherosclerosis [36], elevated plasma levels of inflammatory cytokines [39], and elevated concentrations of free fatty acids, which can contribute to reduced muscle glucose uptake [41] and the development of insulin resistance, which has been directly linked to type 2 diabetes, cardiovascular disease, and cancer [42], [43].

The relationship between stress, eating behavior and obesity during adolescence may be of particular significance given the increased prevalence of obesity in this population. Indeed, in both humans and rodents, the juvenile phase represents a critical period in development during which there is substantial cerebral development and reorganization as well as altered HPA axis function [44], [45]. It has been suggested that this major biological transition period renders adolescents more sensitive to the effects of stressors and the subsequent development of stressor-related psychopathologies [46]. Animal studies have shown unique effects of stressors on HPA activity during adolescence as pre-pubertal rats exhibited higher or prolonged adrenocorticotrophin releasing hormone and corticosterone release compared to adults [44]. However, these effects were sex dependent and also varied with the stressor employed [44]. Adolescence is further characterized as a unique stage in brain development, as regions related to emotional and learning processes, such as the prefrontal cortex, hippocampus, and the amygdala, all undergo substantial remodeling during this phase and have also been proposed to be exquisitely sensitive to the effects of stress [47]. Importantly, these regions have also been implicated in the homeostatic mechanisms underlying energy balance and feeding behaviors [18], [47].

The present study was conducted to further characterize the short- and long-term consequences of stressor exposure during the juvenile period (PD 27–29) on behavioral indices of depression and anxiety using a modified version of the juvenile stress protocol developed previously [48]. A second objective was to determine the effects of juvenile stressors on feeding behavior and preference for a palatable food. In this regard, we aimed to elucidate whether the consumption of a palatable food during adolescence could attenuate the long-term behavioral consequences of stressor exposure. A final objective of this study was to investigate the long term consequences of stress-induced palatable feeding on indices of metabolic syndrome and obesity.

## Materials and Methods

### 1. Animals

Ninety-six 21 day old male Wistar rats were obtained from Charles River (Quebec, Canada). Upon arrival, rats were randomly assigned to four conditions: (1) Chow + Control; (2) Chow + Stress; (3) Palatable + Control; and (4) Palatable + Stress. Rats were double housed until PD 30 or 60 in standard plastic cages with bedding at a room temperature of 22±1°C on a 12 hour light-dark cycle (lights on at 0700hr and off at 1900hr). On PD 30 or 60 all animals were singly housed in order to prepare for social interaction testing. Animals remained singly housed for the remainder of testing. Weight gain was measured on PDs 25, 30, 40, 50, 60, 70. All procedures met the guidelines established by the Canadian Council on Animal Care and were approved by the Animal Care Committee of the University of Ottawa Institute of Mental Health Research.

### 2. Experiments

**Figure 1:**
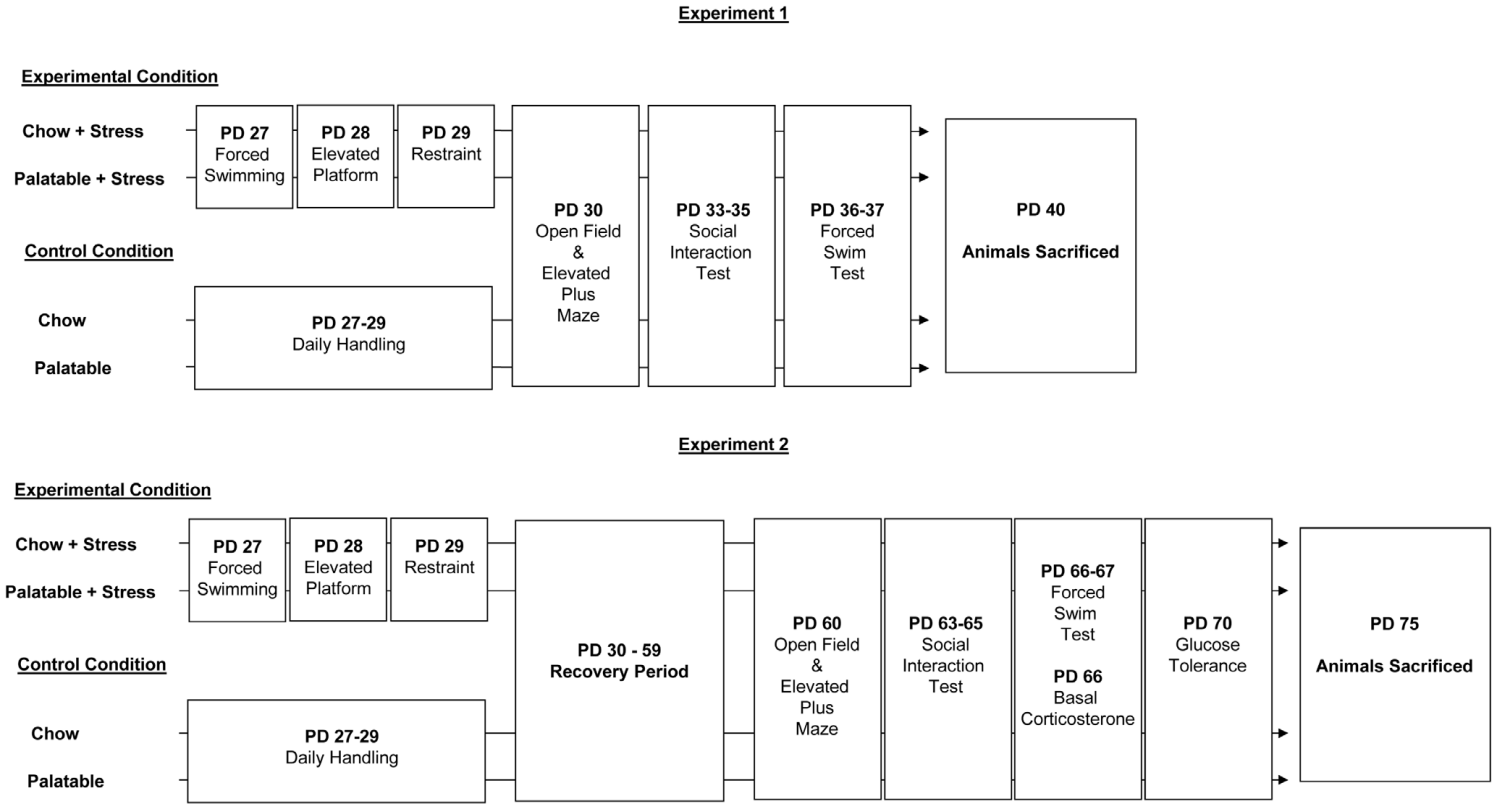
Summary of study design. Animals assigned to stress condition were exposed to a different stressor per day from PD 27–29 while control animals received daily handling. All animals were tested in the indicated behavioral paradigms as described in each experiment.

Forty rats were used in Experiment 1 (juvenile testing, PD-30-37) and fifty-six rats were used in Experiment 2 (adult testing, PD 60-67). Animals in both experiments underwent all behavioral tests. Weight gain, caloric intake, comfort preference, glucose tolerance, plasma corticosterone and adiposity were only collected in Experiment 2. An overview of each experiment is presented in Figure 1.

### 3. Diet

All rats were given free access to standard laboratory chow (3.4 kcal/g, 4.5% fat, 18.1% protein, 57.3% carbohydrate, Charles River Rodent Diet 5075, Agribrand Purina Canada, Woodstock, Ontario, Canada) and rats assigned to the palatable food condition were given limited daily access to a 45%Kcal Fat diet (4.7 Kcal/g, 23.2% fat, 17.3% protein, 47.6% carbohydrates, TD.08811, Harlan Laboratories, Madison, Wisconsin, USA). Palatable food was given daily at 8:00 and removed at 10:00 and intake was measured. Every three days, chow consumption was measured by subtracting the weight of the chow in the cage from its weight 24-hours earlier. Total caloric intake was calculated by multiplying the energy content (Kcal/g) of individual diets by the amount consumed and summing them. Preference for palatable food was calculated by taking the ratio of calories of palatable food consumed over total calories consumed. Food intake data was collected from PD 21 to PD 60. As rats were housed in pairs, equal food intake by cage mates was assumed.

### 4. Juvenile Stress Paradigm

The juvenile stress procedure used was a modification of the 3 day procedure described by Jacobson-Pick and Ritcher-Levin [48]. This procedure comprised 3 consecutive days of exposure to a different stressor per day throughout PD 27–29.

#### PD 27 - Forced swimming

Rats were individually placed in a circular water basin (diameter 48 cm; height 42 cm; water depth 29 cm and a temperature of 22±2°C) for 10 min, during which they swam or floated continuously.

#### PD 28 – Elevated platform

Rats were individually placed on a small elevated platform (12 cm×12 cm; 70 cm elevation from water level) for three separate 30 min sessions. Platforms stood within a basin filled with water for the animals' protection should they fall off. During the intersession interval of 60 min rats were returned to their home cage. Animals which fell during the testing session were immediately returned to the platform for the remainder of the test session.

#### PD 29 – Restraint

Rats were placed for 30 min in a plastic restraining bag that prevented side-to-side movement and limited forward-backward movement. The plastic bag had a hole at the end closest to the rat's nose to allow for ventilation.

### 5. Behavioral Testing

Behavioral testing was conducted under low illumination (30–40 lux) between 10:00–13:00 daily following a 1 hr period during which rats habituated to the testing room. Behavior was monitored via a video camera mounted above the arena. Rats were tested in all behavioral paradigms. Testing was conducted between PD 30–37 or 60–67. An overview of the stress procedure and behavioral testing schedule is presented in Figure 1.

#### 5.1 Open Field

On PD 30 or 60, prior to elevated plus maze (EPM) testing, rats were placed in the center of the arena and its behavior monitored for 5 min. The arena consisted of a square Plexiglas arena measuring 60×60 cm with 30 cm high walls. Using lines, the floor was divided up into 16 squares (5×5 cm). The total number of squares crossed was recorded as an index for general locomotor activity [49].

#### 5.2 Elevated Plus Maze

On PD 30 or 60, rats were placed on the center platform of the EPM. The EPM consisted of two open arms (50×10 cm) and two perpendicularly situated arms enclosed by 40 cm high walls, elevated approximately 66 cm above the floor. Immediately after OF testing, rats were placed onto the open central platform of the EPM (facing a closed arm). Behavior scored during the 5 min test included time spent on the open and closed arms and risk assessment behavior (unprotected head dips; head protruding over the edge of an open arm). Time in the open arms and unprotected head dips are validated measures of reduced anxiety-like behavior [50].

#### 5.3 Social Interaction

The social interaction test was conducted over a total duration of three days. On PD 30 or 60 rats were individually housed 2 hours after the completion of EPM testing in order to increase the level of social interaction [51]. On PD 33 or 63, rats were matched to a partner from the same diet x stress condition (but from a different cage) on the basis of body weight, such that members of a pair did not differ by >10 g. On habituation day 1 (PD 33 or 63), rats along with their test day partner were placed in the arena (60×60 cm; 30 cm high walls) for five minutes. On habituation day 2 (PD 34 or 64), rats were individually placed in the arena for a period of 3 min. On test day (PD 35 or 65), pairs of rats were placed in the test chamber and the behavior of both rats was observed for 7 min. Total time spent in social interaction (including sniffing, climbing over each other, following, allogrooming, and play fighting) was recorded. Decreases in social interaction are reflective of an anxiogenic profile [51].

#### 5.4 Forced Swim Test

The forced swim test is a widely used behavioral despair paradigm used to evaluate the effectiveness of antidepressant drugs [52]–[54]. The forced swim arena consisted of a clear Plexiglas cylinder (20×45 cm, water height 30 cm, temperature 25°C; Stoelting Co., Wood Dale, Illinois). A habituation session (PD 36 or 66: 15 min) was performed twenty-four hours prior to the test session. On test day (PD 37 or 67) the animal was returned to the same cylinder for 5 minutes. The time spent immobile was recorded during the test session.

### 6. Basal Corticosterone Levels and Assay

On PD 66, prior to the FST training session, rats from Experiment 2 were moved in their home cages to the testing room and allowed to rest for a one hour. Blood samples were collected from rats individually using tail venipuncture. The time elapsed from retrieving the rat from their home cage to the depositing of the blood sample on the filter paper was approximately 1–2 min per rat. Blood droplets were deposited onto 903 ProteinSaver filter paper (GE Healthcare Bio-Sciences Corp, MA, USA), allowed to dry at room temperature then stored at −20°C.

Collected samples were analyzed using a radioimmunoassay (RIA). Two days prior to the RIA procedure, blood was eluted from the filter paper by placing one 3 mm punch (per time point) of filter paper in a 12×75 culture tube containing 200 µL Dulbecco's Phosphate Buffered Saline (sigma, item D-5773) w/0.1% gelatine, covered with parafilm in a fridge at 4°C. On the day of the RIA procedure, culture tubes containing the samples were placed on an orbital shaker for 1 hour at room temp. CORT levels were the determined from the eluted blood sample using commercial RIA kits as per the manufacturer's instructions (MP Biomedicals, CA). The inter- and intra-assay variability was 7.3% and 7.4%, respectively.

### 7. Glucose Tolerance

On PD 70, following 12 hr of fasting, rats in Experiment 2 were given an intraperitoneal injection of a 0.75 g/mL dextrose solution (dose: 1.75 g/kg). Blood glucose was measured by applying a drop of blood (via tail venipuncture) onto a test strip, then taking a reading with a blood glucose meter (Accu-Chek Aviva Nano, Roche Diagnostics, Mannheim, Germany). Levels were assessed immediately before the injection, and 15, 30, 60 and 120 min post-injection.

### 8. Adiposity

On PD 75, carcasses of animals in Experiment 2 were collected following sacrifice and then stored at −20°C. The carcasses were later thawed and fat pads hand dissected and weighed. The fat pads collected included: mesenteric, retroperitoneal, subcutaneous inguinal white fat, and inter-scapular brown fat.

### 9. Statistical analysis

Data obtained from the open field, EPM, social interaction, FST, corticosterone and adiposity tests were analyzed by 2 (Diet)×2 (Stress) analysis of variance (ANOVA) for each measure. Data for total caloric intake, palatable food preference, weight, and glucose tolerance were analyzed using three-way (Diet × Stress × Time) repeated-measures ANOVAs where Diet and Stress were the between group variables and Time the (repeated) within group variable. Subsequent follow-up comparisons were conducted using *t* tests with a Bonferroni correction to maintain the alpha level at 0.05. For some variables (weight, caloric intake, palatable food preference, behavioural tests, glucose intolerance and adiposity) a priori predictions that access to the palatable diet would alter the impact of the stressor were made. Follow-up comparisons for interactions for the aforementioned predictions were conducted irrespective of whether the F value for an interaction reached significance [55]. Data points ±3 standard deviations from calculated means were considered as outliers and not included in statistical analysis [56]. Some rats were removed from the statistical analysis of behavioral indices as a result of missing data (e.g. rat fell off the EPM), thus the N and df associated with these measures vary across outcomes.

## Results

### 1. Effect of stress and diet on weight and food intake

**Figure 2:**
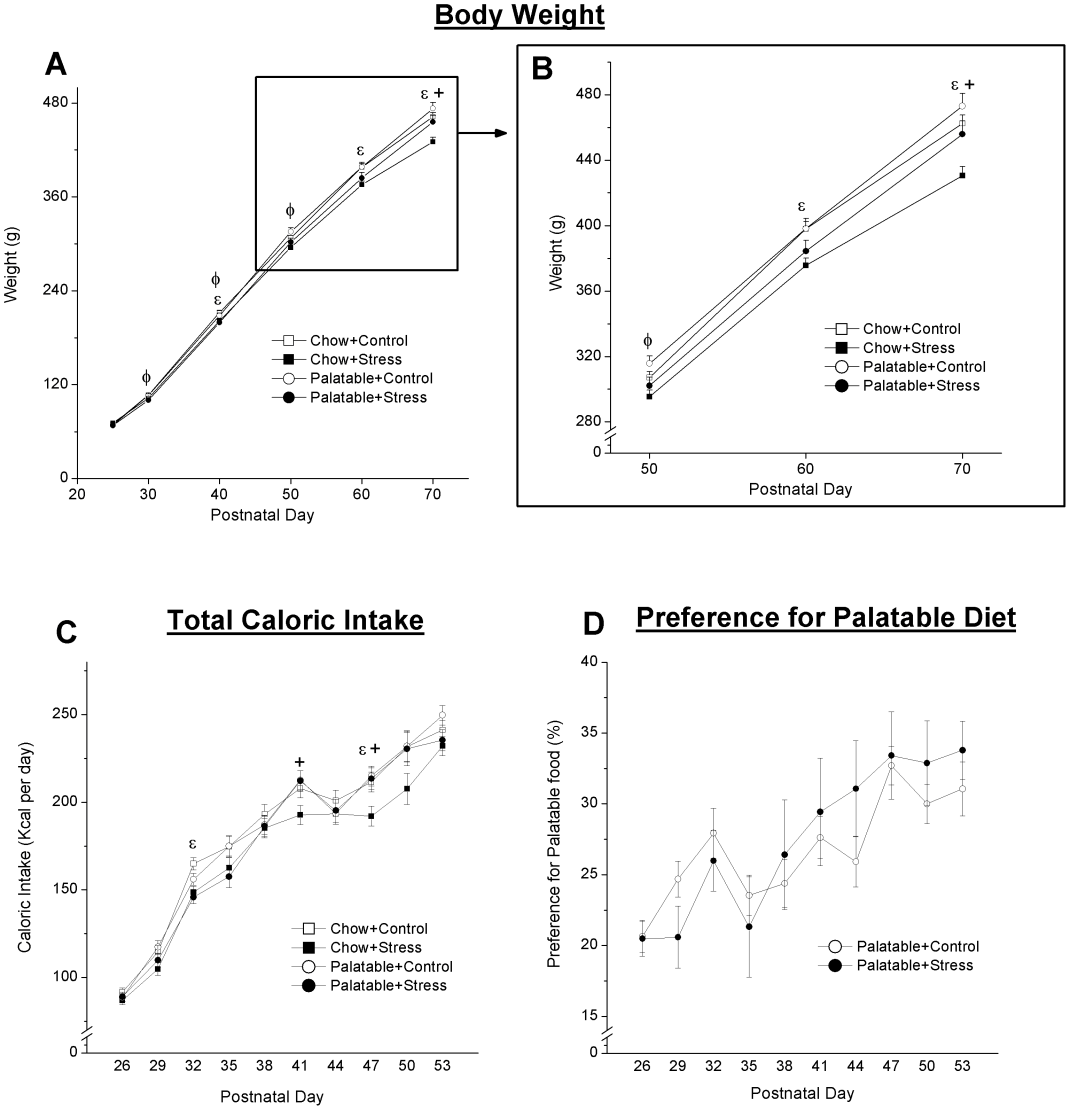
Effect of stress and diet on weight and food intake. Initially, both stress groups gained weight (A and B) at the slower rate relative to their controls; however, this reduction in the rate of weight gain persisted past PD50 in the Chow+Stress group only. A significant difference between stress groups was observed on PD 70. Overall, rats with access to the palatable diet consumed more calories relative to chow-fed rats (C). No significant difference in preference for the palatable diet was observed (D). Lines represent mean ± SEM. ε Significant difference between Chow and Chow+Stress. φ Significant difference between Palatable and Palatable+Stress. + Significant difference between the two stress groups receiving opposite diets.

Juvenile stressor exposure and diet had a significant effect on body weight. Repeated measures ANOVA revealed a significant Stress (F_5,255_ = 7.82, p<.001) and Diet effect (F_5,255_ = 9.89, p<.001) (Figure 2A and 2B). Follow-up comparisons were completed based on an a priori hypothesis that a significant interaction (F_5,255_ = 1.47, p = .201) would be present. Simple effects analysis revealed that previously stressed chow fed rats weighed significantly less than controls at PDs 40 (p = .021), 60 (p = .007) and 70 (p = .002). Among previously stressed rats with access to the palatable diet, a significant decrease in weight relative to the palatable control group was observed on PDs 30 (p = .040), 40 (p = .045), and 50 (p = .033). Among previously stressed rats, those with access to palatable food weighed significantly more than chow fed rats on PD 70 (p = .011).

Repeated measures ANOVA revealed a significant effect of Diet (F_9,207_ = 2.83, p = .026) on total caloric intake (Figure 2C). In general, rats with access to the palatable diet consumed more calories. Follow-up comparisons were completed based on an a priori hypothesis that a significant interaction (F_9,207_ = 1.23, p = .280) would be present. Simple effects analysis showed that previously stressed rats with access to chow only displayed significant reductions in total caloric intake on PD 32 (p = .003) and PD 47 (p = .024). A significant difference between the two stress groups was observed on PD 41 (p = .023) and PD 48 (p = .018). No significant effect of Stress on preference for palatable food (F_9,99_ = 1.895, p = .061; Figure 2D) was observed.

### 2. Effect of stress and diet on anxiety- and depressive-like behavior

Locomotor behavior across groups was comparable in both Experiment 1 and 2. No significant effects of Stress or Diet were found regarding number of squares crossed in the open field at both the juvenile and adult time point. Means (±SEM) for the juvenile time point were as follows: Chow + Control 123.10±14.26; Chow + Stress 118.00±10.25; Palatable + Control 140.10±11.36 and Palatable + Stress 128.10±3.85. Means (±SEM) for the adult time point were as follows: Chow + Control 104.19±6.41; Chow + Stress 98.57±7.81; Palatable + Control 103.85±6.16 and Palatable + Stress 93.86±4.02.

**Figure 3:**
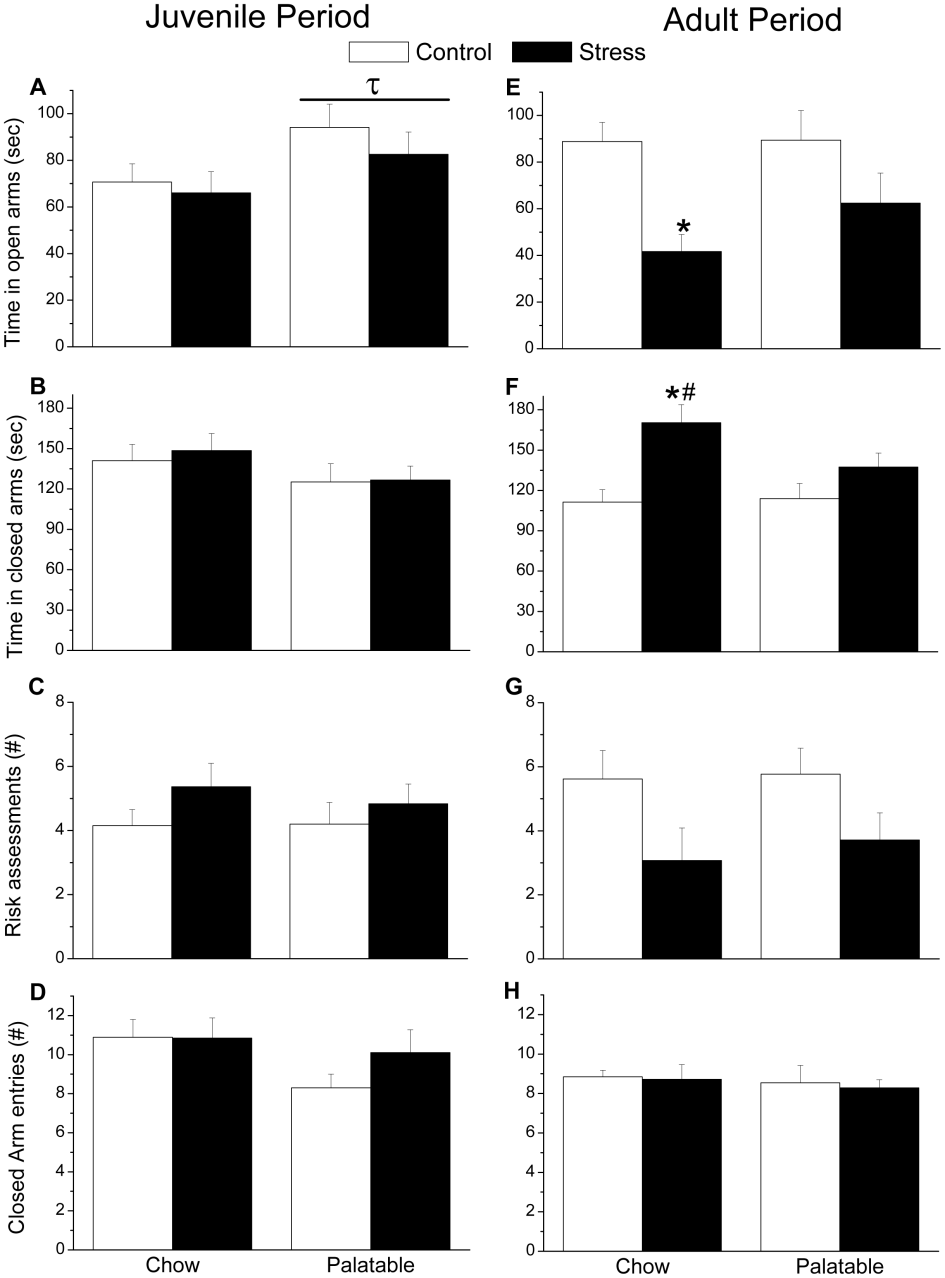
Anxiety-like behavior in the elevated plus maze. A significant effect of the palatable diet was observed in the juvenile time point. In general, rats with access to the palatable diet spent more time in the open arms of the maze. In adulthood, previously stressed chow fed rats (E) spent less time in the open arms, and (F) more time in the closed arms compared to chow fed controls. A significant difference between stress groups was observed in terms of time spent in the close arm with chow-fed rats spending more time relative to palatable food fed counterparts. No significant difference across groups was observed regarding number of (G) risk assessments and closed arms entries (H), suggesting no significant differences in locomotor activity. Bars represent mean ± SEM. τ Significant diet effect. * Significantly different from controls. # Significantly different from condition matched rats receiving opposite diet.

In the EPM (Figure 3, A–D), a significant main effect for Diet on the amount of time spent on the open arms (F_1,35_ = 4.717, p = .037) was observed in Experiment 1. In general, juvenile rats with access to the palatable diet spent more time on the open arms of the maze. No significant differences in time spent in the closed arm, and number of risk assessments was observed. No significant effects of Stress or Diet were found regarding entries into the closed arm suggesting that locomotor activity across groups was comparable.

In Experiment 2, a significant main effects for Stress was observed in the EPM (Figure 3, E–H) for time spent in the open arms (F_1,50_ = 12.271, p = .001), and time spent in the closed arms (F_1,50_ = 13.55, p = .001) suggesting that the juvenile stressor induced long-term anxiety-like behaviors. Follow-up simple effects comparisons on time spent on the open and closed arms were completed based on an a priori hypothesis that significant interactions (F_1,50_ = 2.52, p = .118 for closed; F_1,50_ = .911, p = .345 for open) would be present. Interestingly, previously stressed rats with access to chow spent significantly less time in the open arm (p = .003), and spent more time in the closed arms of the maze (p<.001) compared to their controls. Among the two stress groups, chow fed rats spent significantly more time in the closed arms relative to those with access to the palatable diet (p = .039). No significant differences were observed in terms of the number of risk assessments made and number of entries into the closed arm entries, again suggesting that locomotor activity was comparable across groups.

**Figure 4:**
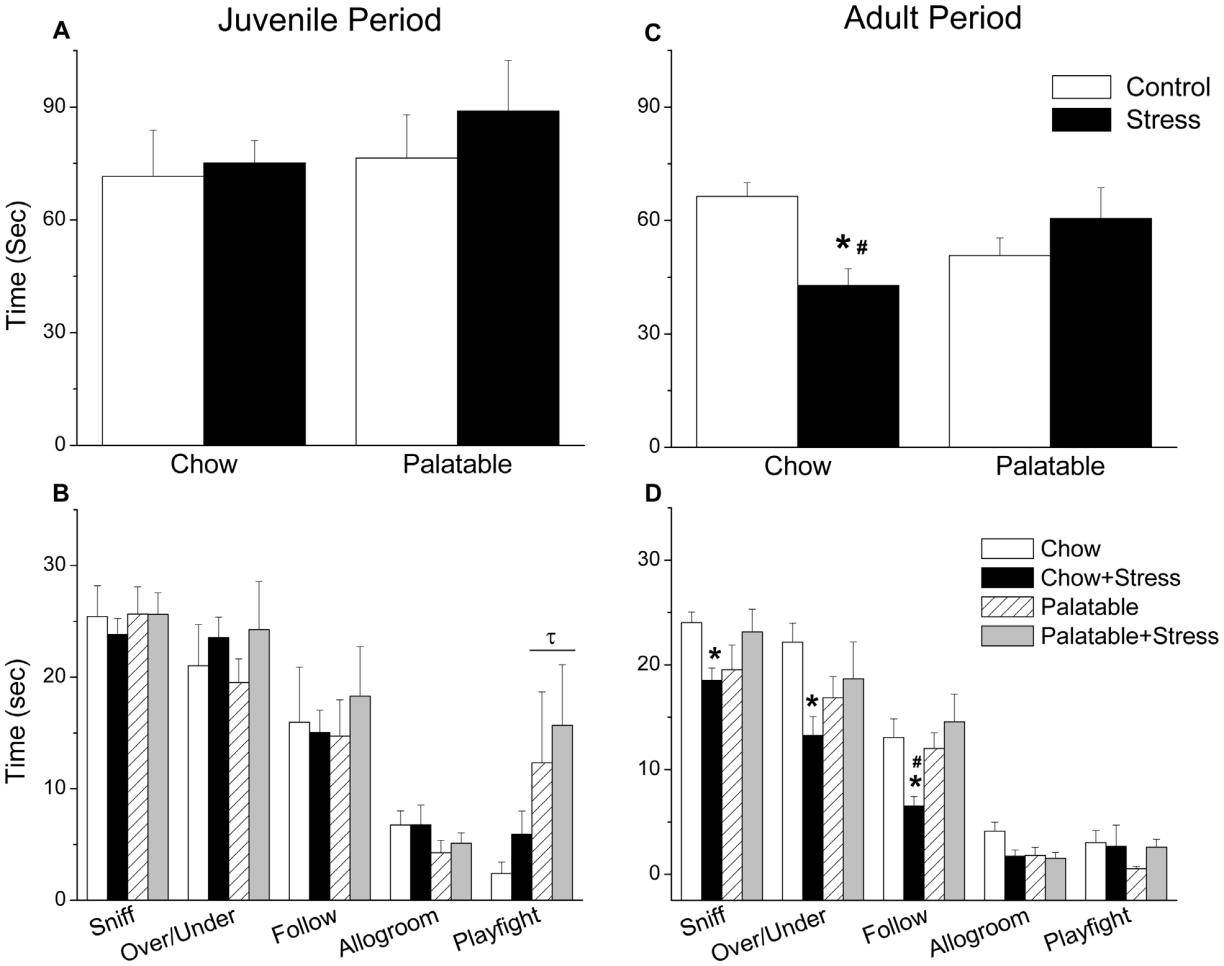
Social behavior in the social interaction test. No significant differences in total time engaged in social behaviors were observed in the juvenile phase (A); however, in general rats with access to the palatable diet engaged in more play fighting (B). In adulthood, previously stressed chow fed rats spent less total time engaged in social behaviors compared to chow-fed controls and previously stressed rats with access to the palatable diet (C). In regards to specific behaviors, chow fed rats exposed to the juvenile stress spent less time sniffing, following and moving over/under one another (D) relative to their controls. The difference between stress groups was significant for following behavior as well (D). Bars represent mean ± SEM. τ Significant diet effect. * Significantly different from controls. # Significantly different from condition matched rats receiving opposite diet.

In the social interaction test, no significant differences in total time spent engaging in active social behaviors was observed in juvenile rats (Figure 4, A–B). In regard to individual social behaviors, a significant Diet effect was observed in time spent play fighting (F_1,35_ = 5.250, p = .028) as rats with access to the palatable diet engaged in more play fighting.

In Experiment 2, juvenile stress induced a long-term increase in anxiety-like behavior measured in the social interaction test (Figure 4, C–D). A significant interaction between Stress and Diet was found with regard to total time spent engaged in social behaviors (F_1,51_ = 9.085, p = .004), following (F_1,51_ = 6.23, p = .016), moving over/under one another (F_1,51_ = 4.93, p = ..031) and time spent sniffing one another (F_1,51_ = 6.41, p = .014). The follow-up simple effects analyses confirmed that previously stressed rats with access to chow spent significantly less time engaged in active social interaction compared to non-stressed controls (p = .004). This decreased activity was also observed in individual social behaviors, including following (p = .015), and moving over/under one another (p = .013) and time spent sniffing on another (p = .037). Stressed rats with access to chow also spent significantly less time total time engaged in social behaviors (p = .029), as well as following one another (p = .003) compared to previously stressed rats with access to palatable food.

For both the juvenile and adult time points, no significant effects of Stress or Diet were found regarding time spent immobile in the FST. Mean (±SEM) time spent immobile for the juvenile time point was as follows: Chow + Control 5.208±1.20; Chow + Stress 3.805±1.20; Palatable + Control 4.060±1.27 and Palatable + Stress 3.298±1.20. Mean (±SEM) time spent immobile for the adult time point was as follows: Chow + Control 21.29±3.96; Chow + Stress 20.89±3.96; Palatable + Control 27.52±4.11 and Palatable + Stress 19.47±4.11.

### 3. Effect of stress and diet on endocrine and metabolic indices

**Figure 5:**
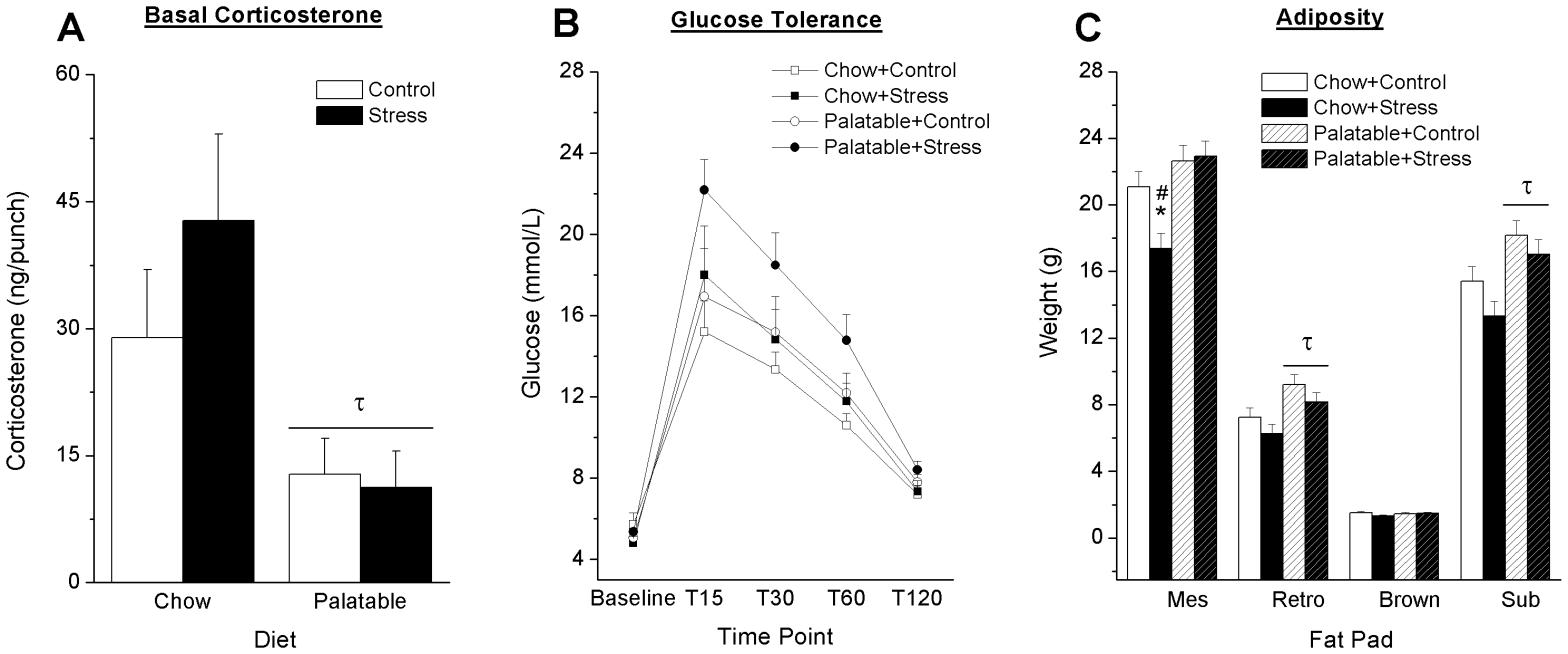
Effect of stress and diet on endocrine and metabolic indices. Rats with access to palatable food displayed lower plasma levels of CORT compared to chow fed rats on PD 66 (A). Blood glucose levels over time following an I.P. injection of a 0.75 g/mL dextrose solution did not differ significantly across groups (B). Rats with access to the palatable diet displayed an increased in retroperitoneal and inguinal subcutaneous fat (C). A decreased weight of the mesenteric fat pad was observed in the Chow+Stress condition relative to their controls and the Palatable+Stress group. Bars represent mean ± SEM. τ Significant diet effect. * Significantly different from controls. ^#^ Significantly different from condition matched rats receiving opposite diet. Mes  =  Mesenteric fat pad. Retro  =  retroperitoneal fat pad. Brown  =  Inter-scapular brown fat. Sub  =  Subcutaneous inguinal white fat pad.

A significant Diet effect was observed in basal corticosterone levels measured on PD 66, F_1,50_ = 10.56, p = .002 (Figure 5A), wherein rats with access to the palatable diet displayed significantly reduced basal corticosterone.

Repeated measures ANOVA revealed a significant Time × Stress interaction for blood glucose levels in response to a glucose challenge, (F_4,200_ = 2.621, p = .036; Figure 5B). Although previously stressed rats with access to the palatable diet tended to show increased blood glucose relative to their controls follow-up simple effects analysis did not reach significance.

In general all rats with access to the palatable diet displayed an increase in adiposity (Figure 5C). A significant Diet effect was observed in the weight of retroperitoneal (F_1,52_ =  10.93 p = .002) and subcutaneous inguinal (F_1,52_ =  10.93 p = .002) fat pads. Two-way ANOVA of mesenteric fat pad weight revealed a significant Diet × Stress interaction (F_1,52_ =  4.75 p = .034). Follow-up simple effects analysis revealed that previously stressed rats with access to chow displayed significant decreases in mesenteric fat relative to previously stressed rats with access to the palatable diet (p<.001), as well as the chow-fed control group (p = .006). No significant differences in inter-scapular brown fat were observed (F_1,52_ =  1.69 p>.1)

## Discussion

The present study investigated the short- and long-term consequences of unpredictable physical stress applied during the juvenile period on subsequent behavioral markers of depression and anxiety and indices of metabolic syndrome and obesity. No short-term effects of juvenile stress on behavioral markers of anxiety-like behaviors were observed in the open field, EPM and social interaction test. In contrast, juvenile stress resulted in decreased exploration of high-risk areas of the EPM in adulthood, which is consistent with results reported by Jacobson-Pick and Ritcher-Levin [48]. Reduced social exploration was also observed in the present study, as rats previously exposed to juvenile stress exhibited less social behavior compared to controls. Decreased social behavior in adulthood following juvenile stress has been previously reported in both mice [57] and rats [58]. Exposure to juvenile stress did not appear to impact behavior in the forced swim test at both time points; however, it is possible that previous exposure to the swim stress in the juvenile stress protocol hay have altered behavior in the FST. This particular stress paradigm may also be more in keeping with a model of anxiety as opposed to depressive symptoms. Together, these findings indicate that juvenile stress can have lasting effects on behavior in adulthood [46], [48], [57]–[62].

Consistent with the view that eating palatable foods may serve as a means of coping with distress in humans [63], [64], in the present study palatable food mitigated the behavioral effects of the juvenile stress. In adulthood, chow-fed rats exposed to the stressor displayed significantly higher levels of anxiety-like behavior compared to rats with access to palatable food. No significant differences occurred in closed arm entries across groups suggesting that the observed results were independent of locomotor activity. Similar results were obtained in the social interaction test with previously stressed chow-fed rats displaying reduced social behavior relative to those with access to the palatable diet. Indeed, access to a palatable diet has been shown to mitigate the long term effects of maternal separation in the sucrose preference test and EPM [35], as well as the effects of short-term restraint stress in adulthood on anxiety-like behavior in the EPM [25]. Similar beneficial effects have been described among adult rats with access to a sucrose solution following a restraint stress [23]. Taken together with these earlier reports, our results provide evidence for the notion of palatable food consumption acts as a buffer against the negative behavioral effects of stress. Our results also provide preliminary evidence that such a diet may have proactive effects that are still evident in adulthood.

While no effects of the stressor were observed when animals were tested as juveniles, a diet effect in the EPM and social interaction test was observed, suggesting that the palatable diet may have had an anxiolytic effect. The lack of a stress effect among chow-fed rats contrasts with previous findings, as stressed juvenile rats were observed to display increased activity and exploration in both the open field and elevated plus maze which the authors characterized as “non-classic anxious behavior” [48; p. 274]. One possible explanation for the observed differences between studies is the modification made to the stressor paradigm. Reducing the duration of restraint stress from 2 hrs in the original protocol to 30 min may have diminished the acute effects of the stressor, resulting in a corresponding change in the behavior in the open field and EPM. Testing windows were also slightly different (24 hours post stress in the current study vs. 1–6 hrs post stress).

Stressors ordinarily increase HPA activity [65], [66] and palatable foods consumption has been associated with lower resting and stress-evoked cortisol levels in humans [67], [68]. In rodents, previous studies have reported normalized HPA activity in adrenalectomized animals [69]–[71], reduced HPA axis response and hypothalamic corticotrophin releasing factor (CRF) mRNA expression following acute restraint stress [34] and diminished HPA axis response to repeated restraint stress [25] when animals are given access to a palatable diet. Moreover, a blunted corticosterone response, as well as reductions in hypothalamic CRF mRNA expression has been reported in response to restraint stress [23]. HPA axis dampening is proposed to be a result of the hedonic properties of the sucrose rather than the increase caloric value, as reductions of plasma corticosterone in response to restraint are not observed when rats were given the same amount and schedule of sucrose solution via intragastric gavage [23]. Consistent with previously reported results, we observed a significant decrease in basal plasma corticosterone concentrations among rats with access to palatable food.

Access to the palatable diet resulted in an overall increase in total caloric intake regardless of stressor exposure. Of greater interest, however, is the proportion of calories obtained from the palatable food to that of the standard chow. As stressor-induced preference for more pleasurable or palatable foods has been documented in both animals and humans [18], [19], [25], we hypothesized that following the 3-day stressor, rats would show a preference for the palatable food. However, despite the mitigating effects of the palatable food on behavioral and neuroendocrine indices of anxiety, no such preference was observed. The lack of preference may be a result of the limited access to the palatable diet, as well as its availability during the light phase. Furthermore, as palatable food was provided in the morning prior to each stressor, it is possible that the timing of access to the palatable food might have influenced our results, as previous research has shown an increase in consumption when the palatable food is provided after the stressor [34]. In addition, palatable preference in the present study was determined on the basis of intake per cage, rather than individual intake as rats were doubly housed. Although this was done to reduce stress stemming from isolation [69]–[71], the result of this decision is a reduction in statistical power, as well as confounding of individual eating patterns. Indeed, previous animal studies documenting a stress-induced increase in palatable feeding provided rats with *ad libitum* access to the favored diet [18], [19], [25].

As expected, juvenile stressor exposure resulted in a decreased rate of weight gain, which is a well-established consequence of stressor exposure in rats [72]. While initially both stress groups displayed a reduction in weight gain, rats with access to the palatable diet displayed a rate of weight gain comparable to their control group from PD 60 onwards, suggesting that access to the palatable food had a mitigating effect on this outcome. This pattern of recovered weight gain has been previously reported following restraint stress [25].

Consistent with findings concerning the negative health consequences of high fat/sugar diets, rats with access to palatable food displayed increased adiposity compared to chow fed rats, despite the absence of any differences in body weight. In addition, previously stressed rats with access to the palatable diet also displayed a significant increase in the mesenteric fat pad compared to the previously stressed chow fed rats. These findings are in line with the perspective that stress alters how fat is distributed throughout the body. It seems that when poor diet is coupled with chronic stress, reorganization of energy stores from peripheral storage to central storage, primarily as abdominal fat, is facilitated, accompanied by elevated levels of glucocorticoids and insulin [19]. High levels of abdominal fat have been associated with hypertension, cardiovascular disease, metabolic syndromes, type 2 diabetes, and morbidity and mortality [73], [74]. However, selectively increased adiposity in the abdominal region is not reported in all studies that examined the effects of palatable food in the rat. Studies investigating the short-term effects of restraint stress [25], [34] and maternal separation [35] demonstrated general increases in fat pad weights among rats with access to palatable foods relative to chow fed rats, but not a selective increases in abdominal fat. Our results may be more germane to the long-term effects of access to a palatable diet. In the present study, rats were sacrificed 6 weeks following termination of the stressor and thus the observed differences in fat distribution between studies might be attributable to the prolonged access to the palatable diet.

High fat diets have been proposed as one of the factors which may lead to reduced insulin sensitivity, followed by insulin resistance and ultimately the development of type 2 diabetes [3]. This has a corresponding effect on the ability of the body to process glucose. Reduced ability to tolerate a glucose load was not observed in the present study; however, the development of glucose intolerance following access to a high fat diet has been previously reported [75]–[78]. Exposure to a stressor may also contribute to the development of glucose intolerance. Dysregulation of the HPA axis as a result of stressor exposure, in conjunction with chronically elevated insulin levels, contribute to the development of insulin resistance, abdominal obesity, as well as metabolic syndrome [79].

## Conclusion

The present study provides additional evidence that stressor exposure during juvenility can have long lasting effects on anxiety-like behaviors in adulthood, and that access to palatable food may have mitigating effects on the anxiety and corticosterone effects of juvenile stress. Not only do these results provide further support for the notion that palatable foods may be protective against the negative effects of stress, but also that these effects last into adulthood. However, the use of palatable foods as coping-strategy has long term negative effects on adiposity.

## References

[1] International Obesity Task Force (2010) Obesity: The Global Epidemic. http://www.iaso.org/iotf/obesity/obesitytheglobalepidemic/. Acessed 2013 November 1.

[2] Singh AS, Mulder C, Twisk JW, van Mechelen W, Chinapaw MJ (2008) Tracking of childhood overweight into adulthood: a systematic review of the literature. Obes Rev 9: 474–488. 18331423 10.1111/j.1467-789X.2008.00475.x

[3] Spriijt-Metz D (2011) Etiology, treatment, and prevention of obesity in childhood and adolescence: A decade in review. Journal of Reserach on Adolescence 21: 129–152. 10.1111/j.1532-7795.2010.00719.x PMC3102537 21625328

[4] Whitaker RC, Wright JA, Pepe MS, Seidel KD, Dietz WH (1997) Predicting obesity in young adulthood from childhood and parental obesity. N Engl J Med 337: 869–873. 9302300 10.1056/NEJM199709253371301

[5] Shaibi GQ, Goran MI (2008) Examining metabolic syndrome definitions in overweight Hispanic youth: a focus on insulin resistance. J Pediatr 152: 171–176. 18206684 10.1016/j.jpeds.2007.08.010 PMC2474653

[6] Freedman DS, Dietz WH, Srinivasan SR, Berenson GS (1999) The relation of overweight to cardiovascular risk factors among children and adolescents: the Bogalusa Heart Study. Pediatrics 103: 1175–1182. 10353925 10.1542/peds.103.6.1175

[7] Pinhas-Hamiel O, Zeitler P (1996) Insulin resistance, obesity, and related disorders among black adolescents. J Pediatr 129: 319–320. 8804318 10.1016/s0022-3476(96)70060-4

[8] Cruz ML, Shaibi GQ, Weigensberg MJ, Spruijt-Metz D, Ball GD, et al (2005) Pediatric obesity and insulin resistance: chronic disease risk and implications for treatment and prevention beyond body weight modification. Annu Rev Nutr 25: 435–468. 16011474 10.1146/annurev.nutr.25.050304.092625

[9] Calle EE, Thun MJ (2004) Obesity and cancer. Oncogene 23: 6365–6378. 15322511 10.1038/sj.onc.1207751

[10] Calle EE, Kaaks R (2004) Overweight, obesity and cancer: epidemiological evidence and proposed mechanisms. Nat Rev Cancer 4: 579–591. 15286738 10.1038/nrc1408

[11] Hills AP, Andersen LB, Byrne NM (2011) Physical activity and obesity in children. Br J Sports Med 45: 866–870. 21836171 10.1136/bjsports-2011-090199

[12] Coccurello R, D'Amato FR, Moles A (2009) Chronic social stress, hedonism and vulnerability to obesity: lessons from rodents. Neurosci Biobehav Rev 33: 537–550. 18585781 10.1016/j.neubiorev.2008.05.018

[13] Hill JO, Peters JC (1998) Environmental contributions to the obesity epidemic. Science 280: 1371–1374. 9603719 10.1126/science.280.5368.1371

[14] Torres SJ, Nowson CA (2007) Relationship between stress, eating behavior, and obesity. Nutrition 23: 887–894. 17869482 10.1016/j.nut.2007.08.008

[15] Zellner DA, Loaiza S, Gonzalez Z, Pita J, Morales J, et al (2006) Food selection changes under stress. Physiol Behav 87: 789–793. 16519909 10.1016/j.physbeh.2006.01.014

[16] O'Connor DB, Jones F, Conner M, McMillan B, Ferguson E (2008) Effects of daily hassles and eating style on eating behavior. Health Psychol 27: S20–S31. 18248102 10.1037/0278-6133.27.1.S20

[17] Gibson EL (2006) Emotional influences on food choice: sensory, physiological and psychological pathways. Physiol Behav 89: 53–61. 16545403 10.1016/j.physbeh.2006.01.024

[18] Dallman MF (2010) Stress-induced obesity and the emotional nervous system. Trends Endocrinol Metab 21: 159–165. 19926299 10.1016/j.tem.2009.10.004 PMC2831158

[19] Dallman MF, Pecoraro NC, la Fleur SE (2005) Chronic stress and comfort foods: self-medication and abdominal obesity. Brain Behav Immun 19: 275–280. 15944067 10.1016/j.bbi.2004.11.004

[20] Dess NK (1992) Divergent responses to saccharin vs. sucrose availability after stress in rats. Physiol Behav 52: 115–125. 1528993 10.1016/0031-9384(92)90440-d

[21] Young JB (2000) Effects of neonatal handling on sympathoadrenal activity and body composition in adult male rats. Am J Physiol Regul Integr Comp Physiol 279: R1745–R1752. 11049858 10.1152/ajpregu.2000.279.5.R1745

[22] Ulrich-Lai YM, Ostrander MM, Thomas IM, Packard BA, Furay AR, et al (2007) Daily limited access to sweetened drink attenuates hypothalamic-pituitary-adrenocortical axis stress responses. Endocrinology 148: 1823–1834. 17204558 10.1210/en.2006-1241 PMC4408907

[23] Ulrich-Lai YM, Christiansen AM, Ostrander MM, Jones AA, Jones KR, et al (2010) Pleasurable behaviors reduce stress via brain reward pathways. Proc Natl Acad Sci U S A 107: 20529–20534. 21059919 10.1073/pnas.1007740107 PMC2996660

[24] Ulrich-Lai YM, Ostrander MM, Herman JP (2011) HPA axis dampening by limited sucrose intake: reward frequency vs. caloric consumption. Physiol Behav 103: 104–110. 21168428 10.1016/j.physbeh.2010.12.011 PMC3056892

[25] Pecoraro N, Reyes F, Gomez F, Bhargava A, Dallman MF (2004) Chronic stress promotes palatable feeding, which reduces signs of stress: feedforward and feedback effects of chronic stress. Endocrinology 145: 3754–3762. 15142987 10.1210/en.2004-0305

[26] la Fleur SE, Houshyar H, Roy M, Dallman MF (2005) Choice of lard, but not total lard calories, damps adrenocorticotropin responses to restraint. Endocrinology 146: 2193–2199. 15705773 10.1210/en.2004-1603

[27] Kant GJ, Bauman RA (1993) Effects of chronic stress and time of day on preference for sucrose. Physiol Behav 54: 499–502. 8415943 10.1016/0031-9384(93)90242-8

[28] Fachin A, Silva RK, Noschang CG, Pettenuzzo L, Bertinetti L, et al (2008) Stress effects on rats chronically receiving a highly palatable diet are sex-specific. Appetite 51: 592–598. 18524415 10.1016/j.appet.2008.04.016

[29] Christiansen AM, Dekloet AD, Ulrich-Lai YM, Herman JP (2011) “Snacking” causes long term attenuation of HPA axis stress responses and enhancement of brain FosB/deltaFosB expression in rats. Physiol Behav 103: 111–116. 21262247 10.1016/j.physbeh.2011.01.015 PMC3060034

[30] Buwalda B, Blom WA, Koolhaas JM, van Dijk G (2001) Behavioral and physiological responses to stress are affected by high-fat feeding in male rats. Physiol Behav 73: 371–377. 11438364 10.1016/s0031-9384(01)00493-0

[31] Zeeni N, Daher C, Fromentin G, Tome D, Darcel N, et al (2012) A cafeteria diet modifies the response to chronic variable stress in rats. Stress 16: 211–219. 22775984 10.3109/10253890.2012.708952

[32] Strack AM, Akana SF, Horsley CJ, Dallman MF (1997) A hypercaloric load induces thermogenesis but inhibits stress responses in the SNS and HPA system. Am J Physiol 272: R840–R848. 9087645 10.1152/ajpregu.1997.272.3.R840

[33] Levin BE (1996) Reduced paraventricular nucleus norepinephrine responsiveness in obesity-prone rats. Am J Physiol 270: R456–R461. 8779879 10.1152/ajpregu.1996.270.2.R456

[34] Foster MT, Warne JP, Ginsberg AB, Horneman HF, Pecoraro NC, et al (2009) Palatable foods, stress, and energy stores sculpt corticotropin-releasing factor, adrenocorticotropin, and corticosterone concentrations after restraint. Endocrinology 150: 2325–2333. 19106219 10.1210/en.2008-1426 PMC2671911

[35] Maniam J, Morris MJ (2010) Palatable cafeteria diet ameliorates anxiety and depression-like symptoms following an adverse early environment. Psychoneuroendocrinology 35: 717–728. 19939573 10.1016/j.psyneuen.2009.10.013

[36] de Ferranti S, Mozaffarian D (2008) The perfect storm: obesity, adipocyte dysfunction, and metabolic consequences. Clin Chem 54: 945–955. 18436717 10.1373/clinchem.2007.100156

[37] Woods SC, Seeley RJ (2000) Adiposity signals and the control of energy homeostasis. Nutrition 16: 894–902. 11054594 10.1016/s0899-9007(00)00454-8

[38] Kriketos AD, Greenfield JR, Peake PW, Furler SM, Denyer GS, et al (2004) Inflammation, insulin resistance, and adiposity: a study of first-degree relatives of type 2 diabetic subjects. Diabetes Care 27: 2033–2040. 15277436 10.2337/diacare.27.8.2033

[39] Bastard JP, Maachi M, Lagathu C, Kim MJ, Caron M, et al (2006) Recent advances in the relationship between obesity, inflammation, and insulin resistance. Eur Cytokine Netw 17: 4–12. 16613757

[40] Korner J, Leibel RL (2003) To eat or not to eat - how the gut talks to the brain. N Engl J Med 349: 926–928. 12954739 10.1056/NEJMp038114

[41] Randle PJ (1998) Regulatory interactions between lipids and carbohydrates: the glucose fatty acid cycle after 35 years. Diabetes Metab Rev 14: 263–283. 10095997 10.1002/(sici)1099-0895(199812)14:4<263::aid-dmr233>3.0.co;2-c

[42] Jee SH, Kim HJ, Lee J (2005) Obesity, insulin resistance and cancer risk. Yonsei Med J 46: 449–455. 16127767 10.3349/ymj.2005.46.4.449 PMC2815827

[43] DeFronzo RA, Ferrannini E (1991) Insulin resistance. A multifaceted syndrome responsible for NIDDM, obesity, hypertension, dyslipidemia, and atherosclerotic cardiovascular disease. Diabetes Care 14: 173–194. 2044434 10.2337/diacare.14.3.173

[44] McCormick CM, Mathews IZ (2010) Adolescent development, hypothalamic-pituitary-adrenal function, and programming of adult learning and memory. Prog Neuropsychopharmacol Biol Psychiatry 34: 756–765. 19782715 10.1016/j.pnpbp.2009.09.019

[45] Bingham B, Gray M, Sun T, Viau V (2011) Postnatal blockade of androgen receptors or aromatase impair the expression of stress hypothalamic-pituitary-adrenal axis habituation in adult male rats. Psychoneuroendocrinology 36: 249–257. 20719434 10.1016/j.psyneuen.2010.07.015

[46] Avital A, Richter-Levin G (2005) Exposure to juvenile stress exacerbates the behavioural consequences of exposure to stress in the adult rat. Int J Neuropsychopharmacol 8: 163–173. 15546500 10.1017/S1461145704004808

[47] Spear LP (2000) The adolescent brain and age-related behavioral manifestations. Neurosci Biobehav Rev 24: 417–463. 10817843 10.1016/s0149-7634(00)00014-2

[48] Jacobson-Pick S, Richter-Levin G (2010) Differential impact of juvenile stress and corticosterone in juvenility and in adulthood, in male and female rats. Behav Brain Res 214: 268–276. 20561965 10.1016/j.bbr.2010.05.036

[49] Correa M, Arizzi MN, Betz A, Mingote S, Salamone JD (2003) Open field locomotor effects in rats after intraventricular injections of ethanol and the ethnaol metabolites acetaldehyde and acetate. Br Res Bull 62: 197–202. 10.1016/j.brainresbull.2003.09.013 14698353

[50] Carobrez AP, Bertoglio LJ (2005) Ethological and temporal analyses of anxiety-like behavior: the elevated plus-maze model 20 years on. Neurosci Biobehav Rev 29: 1193–1205. 16084592 10.1016/j.neubiorev.2005.04.017

[51] File SE, Seth P (2003) A review of 25 years of the social interaction test. Eur J Pharmacol 463: 35–53. 12600701 10.1016/s0014-2999(03)01273-1

[52] Schiller GD, Pucilowski O, Wienicke C, Overstreet DH (1992) Immobility-reducing effects of antidepressants in a genetic animal model of depression. Brain Res Bull 28: 821–823. 1617465 10.1016/0361-9230(92)90267-2

[53] Porsolt RD, Bertin A, Jalfre M (1977) Behavioral despair in mice: a primary screening test for antidepressants. Arch Int Pharmacodyn Ther 229: 327–336. 596982

[54] Porsolt RD, Anton G, Blavet N, Jalfre M (1978) Behavioural despair in rats: a new model sensitive to antidepressant treatments. Eur J Pharmacol 47: 379–391. 204499 10.1016/0014-2999(78)90118-8

[55] Winer BJ (1962) Statistical Principles in Experimental Design. New York: McGraw-Hill.

[56] Taylor JR (1997) An Introduction to Error Analysis. Sausolito, California: University Science Books.

[57] Jacobson-Pick S, Audet MC, Nathoo N, Anisman H (2011) Stressor experiences during the juvenile period increase stressor responsivity in adulthood: transmission of stressor experiences. Behav Brain Res 216: 365–374. 20732357 10.1016/j.bbr.2010.08.016

[58] Toth E, Avital A, Leshem M, Richter-Levin G, Braun K (2008) Neonatal and juvenile stress induces changes in adult social behavior without affecting cognitive function. Behav Brain Res 190: 135–139. 18367262 10.1016/j.bbr.2008.02.012

[59] Avital A, Ram E, Maayan R, Weizman A, Richter-Levin G (2006) Effects of early-life stress on behavior and neurosteroid levels in the rat hypothalamus and entorhinal cortex. Brain Res Bull 68: 419–424. 16459196 10.1016/j.brainresbull.2005.09.015

[60] Jacobson-Pick S, Elkobi A, Vander S, Rosenblum K, Richter-Levin G (2008) Juvenile stress-induced alteration of maturation of the GABAA receptor alpha subunit in the rat. Int J Neuropsychopharmacol 11: 891–903. 18364065 10.1017/S1461145708008559

[61] Taylor SE, Klein LC, Lewis BP, Gruenewald TL, Gurung RA, et al (2000) Biobehavioral responses to stress in females: tend-and-befriend, not fight-or-flight. Psychol Rev 107: 411–429. 10941275 10.1037/0033-295x.107.3.411

[62] Toledo-Rodriguez M, Sandi C (2007) Stress before puberty exerts a sex- and age-related impact on auditory and contextual fear conditioning in the rat. Neural Plast 2007: 71203. 17671613 10.1155/2007/71203 PMC1931496

[63] Dube L, LeBel JL, Lu J (2005) Affect asymmetry and comfort food consumption. Physiol Behav 86: 559–567. 16209880 10.1016/j.physbeh.2005.08.023

[64] Macht M (2008) How emotions affect eating: a five-way model. Appetite 50: 1–11. 17707947 10.1016/j.appet.2007.07.002

[65] Greaves-Lord K, Ferdinand RF, Oldehinkel AJ, Sondeijker FE, Ormel J, et al (2007) Higher cortisol awakening response in young adolescents with persistent anxiety problems. Acta Psychiatr Scand 116: 137–144. 17650276 10.1111/j.1600-0447.2007.01001.x

[66] Kallen VL, Tulen JH, Utens EM, Treffers PD, De Jong FH, et al (2008) Associations between HPA axis functioning and level of anxiety in children and adolescents with an anxiety disorder. Depress Anxiety 25: 131–141. 17340603 10.1002/da.20287

[67] Deuster PA, Singh A, Hofmann A, Moses FM, Chrousos GC (1992) Hormonal responses to ingesting water or a carbohydrate beverage during a 2 h run. Med Sci Sports Exerc 24: 72–79. 1549000

[68] Markus R, Panhuysen G, Tuiten A, Koppeschaar H (2000) Effects of food on cortisol and mood in vulnerable subjects under controllable and uncontrollable stress. Physiol Behav 70: 333–342. 11006432 10.1016/s0031-9384(00)00265-1

[69] Einon DF, Morgan MJ (1977) A critical period for social isolation in the rat. Dev Psychobiol 10: 123–132. 838157 10.1002/dev.420100205

[70] Fone KCF, Dixon DM (1991) Acute and chronic effects of intrathecal galanin on behavioural and biochemical markers of spinal motor function in adult rats. Brain Res 544: 118–125. 1713112 10.1016/0006-8993(91)90892-y

[71] Weiss IC, Pryce CR, Jongen-Relo AL, Nanz-Bahr NI, Feldon J (2004) Effect of social isolation on stress-related behavioural and neuroendocrine state in the rat. Behav Brain Res 152: 279–295. 15196796 10.1016/j.bbr.2003.10.015

[72] Marti O, Gavalda A, Jolin T, Armario A (1996) Acute stress attenuates but does not abolish circadian rhythmicity of serum thyrotrophin and growth hormone in the rat. Eur J Endocrinol 135: 703–708. 9025716 10.1530/eje.0.1350703

[73] Stunkard AJ, Faith MS, Allison KC (2003) Depression and obesity. Biol Psychiatry 54: 330–337. 12893108 10.1016/s0006-3223(03)00608-5

[74] Friedman JM (2003) A war on obesity, not the obese. Science 299: 856–858. 12574619 10.1126/science.1079856

[75] Garg N, Thakur S, Alex MC, Adamo ML (2011) High fat diet induced insulin resistance and glucose intolerance are gender-specific in IGF-1R heterozygous mice. Biochem Biophys Res Commun 412: 476–480. 10.1016/j.bbrc.2011.08.123 PMC3185216 21910970

[76] Cerf ME (2007) High fat diet modulation of glucose sensing in the beta-cell. Med Sci Monit 13: RA12–RA17. 17179917

[77] Akyol A, McMullen S, Langley-Evans SC (2012) Glucose intolerance associated with early-life exposure to maternal cafeteria feeding is dependent upon post-weaning diet. Br J Nutr 107: 964–978. 21861941 10.1017/S0007114511003916

[78] Akerfeldt MC, Laybutt DR (2011) Inhibition of Id1 Augments Insulin Secretion and Protects Against High-Fat Diet-Induced Glucose Intolerance. Diabetes 60: 2506–2514. 21940780 10.2337/db11-0083 PMC3178288

[79] Pervanidou P, Chrousos GP (2011) Stress and obesity/metabolic syndrome in childhood and adolescence. Int J Pediatr Obes 6 Suppl 1 21–28. 10.3109/17477166.2011.615996 21905812

